# GraphQL for the delivery of bioinformatics web APIs and application to ZincBind

**DOI:** 10.1093/bioadv/vbab023

**Published:** 2021-09-29

**Authors:** Sam M Ireland, Andrew C R Martin

**Affiliations:** Division of Biosciences, Institute of Structural and Molecular Biology, University College London, London WC1E 6BT, UK

## Abstract

**Motivation:**

Many bioinformatics resources are provided as ‘web services’, with large databases and analysis software stored on a central server, and clients interacting with them using the hypertext transport protocol (HTTP). While some provide only a visual HTML interface, requiring a web browser to use them, many provide programmatic access using a web application programming interface (API) which returns XML, JSON or plain text that computer programs can interpret more easily. This allows access to be automated. Initially, many bioinformatics APIs used the ‘simple object access protocol’ (SOAP) and, more recently, representational state transfer (REST).

**Results:**

GraphQL is a novel, increasingly prevalent alternative to REST and SOAP that represents the available data in the form of a graph to which any conceivable query can be submitted, and which is seeing increasing adoption in industry. Here, we review the principles of GraphQL, outline its particular suitability to the delivery of bioinformatics resources and describe its implementation in our ZincBind resource.

**Availability and implementation:**

https://api.zincbind.net.

**Supplementary information:**

Supplementary data are available at *Bioinformatics Advances* online.

## 1 Introduction

Over the past three decades, there has been a proliferation of biological datasets, as well as software for the analysis of these data. Advances in many disparate areas of biology depend on access to these resources which are often located in central data stores managed by large institutions, with researchers submitting data to these central repositories. Organizations, such as the European Bioinformatics Institute (EBI), the National Centre for Biotechnology Information (NCBI) and the Research Collaboratory for Structural Bioinformatics (RCSB), maintain large databanks, databases and suites of software for processing biological data. The software is often distributed as source code or compiled binaries, but to use software with the relevant data, (portions of) those datasets need to be on the same machine as the software, meaning that large datasets may need to be moved to the researcher’s local machine.

Thus, one of the central problems of bioinformatics is how to make these datasets accessible to the research community without researchers needing to maintain local copies (Stein, 2002). How can researchers analyze data when the software performing the analysis is located on a different machine? Early approaches made use of the maturing internet to use grid-based, peer-to-peer solutions which were effective over local networks but did not scale well to the level of the entire internet (Neerincx and Leunissen, 2005). This resulted in a proliferation of competing protocols and standards making it tedious and difficult to combine services into a single pipeline—the so-called ‘interoperability problem’.

The web—which uses the simple hypertext transport protocol (HTTP) for transferring information in the form of connected documents (HTML, initially)—became ubiquitous in the 1990s, which led to an initial solution to this problem. Since most researchers had access to a web browser, the HTTP protocol (and more recently its encrypted variant, HTTPS) became the standard transport mechanism, and bioinformatics service providers created web interfaces to their datasets and tools—HTML views and forms that could be accessed with a browser. Users could therefore interact with these central data stores and software using these interfaces.

While this was a vast improvement, it did not solve the interoperability problem—the services were standalone web interfaces, with no ability to communicate with each other or be chained together into more useful pipelines. The collection of data from these resources could only be done using ‘web scraping’ (or ‘screen scraping’): scripts that would download and process the HTML to extract relevant information. This task could be eased by using ‘semantic markup’ of the HTML (i.e. semantically meaningful ‘class’ or ‘id’ attributes in the markup), but generally, this was not done and a change in the HTML display would break such scrapers. With the advent of more complex websites, where more of the data display logic is performed within the web browser using JavaScript rather than on the server, screen scraping becomes almost impossible.

The solution was to provide a web application programming interface (API), served over HTTP, but with responses that are easily machine parsable. This allows local computer programs to communicate with databases and software on central servers in an automated manner, and for a single local computer program to acquire and process data from multiple services without laborious manual human intervention.

After a brief exploration of CORBA, the earliest well-established standard used by bioinformatics APIs was SOAP (Simple Object Access Protocol). SOAP packages function calls and data using XML, and allows one to write code that calls a function as if it were available locally, while it is actually present on a remote server. The server can have a schema to describe the format of messages it expects and returns using an XML format known as WSDL (Web Service Definition Language), which allows the client to validate requests before sending. Together with UDDI (Universal Description, Discovery and Integration—a specification for a distributed registry of web services), this allowed client software to discover and use web services without the programmer knowing where they are or how they are called. Many of the early bioinformatics web APIs used SOAP and, by 2005, included those provided by the EBI (Pillai *et al.*, 2005). The RCSB started work on a similar service the same year (Deshpande, 2005), and many other smaller resources developed SOAP interfaces around the same time.

This went a long way toward solving the interoperability problem, but while SOAP was popular in the 2000s, it suffered from rigidity and, despite the word ‘simple’ in the name, was over complex. It required the client to generate and properly format an XML message in a specific way, meaning each programming language needed its own SOAP library. It was also somewhat complex to develop and maintain from the server-side perspective, and its XML responses tended to be needlessly large.

REST (REpresentational State Transfer) is a very simple alternative to SOAP that was proposed in 2000 (Fielding, 2000), and had largely replaced SOAP by the early 2010s. Rather than complex packaging of data and requests using XML, a REST API simply accepts HTTP requests and returns HTTP responses—but responses containing computer parsable data rather than HTML. The client indicates what it wants using HTTP verbs (GET, POST, DELETE, etc.) and by specifying the location of the resource it wishes to access using a URI such as ‘/gene?id=100’. Since this uses existing HTTP concepts, it requires no specialized ‘REST library’ for the client, only a standard HTTP library of the kind that is often in the standard library of modern programming languages. The API exposes a number of endpoints corresponding to the different resources, and the client makes requests to whatever endpoint it requires. Responses can be in any machine-readable form, such as keyword/value pairs, but are typically XML or, more commonly, JSON (JavaScript Object Notation).

By 2010, many large bioinformatics institutions were offering REST services alongside their earlier SOAP interface (Lopez *et al.*, 2014; Rose *et al.*, 2011) and, over the course of the 2010s, many of these SOAP APIs were deprecated and removed (Yates *et al.*, 2015). By 2020, REST became the dominant style for bioinformatics web APIs.

### 1.1 GraphQL—a better web API standard

GraphQL is an alternative architecture for web APIs. In a GraphQL API, as with SOAP, a single endpoint is exposed, typically ‘/graphql’. The client sends HTTP requests to this endpoint and specifies what it requires, not through the choice of URI or HTTP verb (as is done with REST), but by sending a GraphQL message in the request body, structured in a particular way, which describes *precisely* what data it would like.

**Fig. 1.**
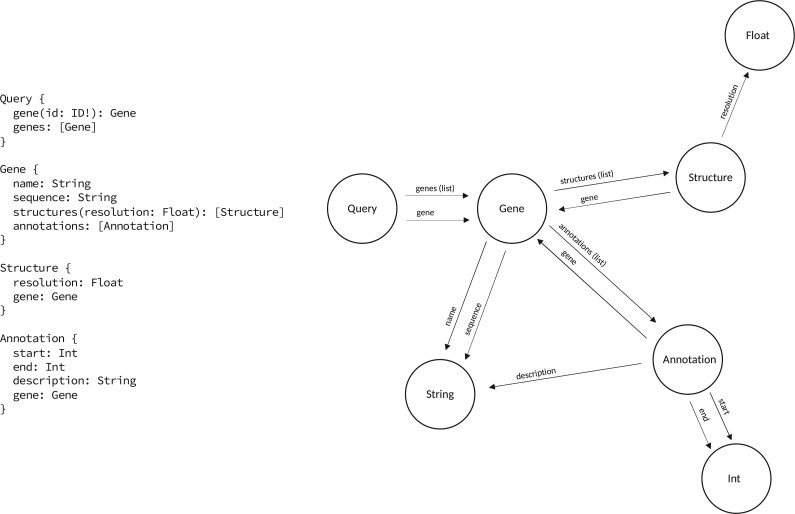
An illustration of a simple bioinformatics GraphQL schema and its representation as a graph. On the left, the schema is shown in the standard GraphQL schema format. Here, the four object types are shown, with their name and the fields each object has (along with any arguments those fields might have in brackets). In this notation, if the field type is enclosed in square brackets, it signifies that the field represents an array (or list) of the specified type, not a single object. On the right, this same schema is shown in graph form, including the primitive types (those without fields, shaded in grey). Here, the nodes are the types and the edges are the fields that link those object types. The Query object is required in all GraphQL schemas and acts as the starting point for all GraphQL query requests

A GraphQL backend is defined with a schema, which is a list of ‘types’. These can be primitive types, such as integer, string, etc., or object types that have attributes or ‘fields’. These fields also have a type, which must correspond to a type elsewhere in the schema. For example, the schema might contain the ‘Gene’ object type, with a field for ID (integer type), name (string type) and annotations (a list of Annotation types). The schema can therefore be viewed as a graph, with the types acting as nodes and their fields the edges between the nodes as illustrated in Figure 1. All schemas have a Query object which acts as a starting point; this Query object has fields that give access to other types, from which the rest of the graph is accessible.

The task of taking a valid GraphQL query and actually populating it with data is done by functions called resolvers, which are written by the developer and, in simple cases, such as this, simply need to fetch data from a database, though any logic is possible here. For example, one can use additional fields not present in the database, but which are calculated from database fields during the execution of the resolver function, or modify the database values before sending them.

Fields, the edges between object types, can represent a one-to-one relationship or a many-to-one relationship. For example, a ‘gene’ object type might have a many-to-one field called ‘annotations’ which points to the ‘annotation’ object type, signifying that genes can have multiple annotations associated. Conversely, the ‘annotation’ object type might have a one-to-one field ‘gene’, signifying that an annotation object will be associated with one gene object. In the JSON output, this is represented as a list of objects. A common, slightly more complex pattern, is to have a specific ‘edge’ object type that represents the list, and which itself has the many-to-one relationship with the object type in question. So there might be an ‘AnnotationEdge’ object, which represents a collection of annotations, and which will have a many-to-one field for the list of annotations themselves. This allows the edges in the graph to have properties themselves (‘count’ being a useful and very common example) that can be requested without needing to request the list of objects itself. This pattern is not part of the GraphQL specification itself, but is widely used when representing large datasets. See the ZincBindDB schema in the Supplementary Materials for a practical example.

**Fig. 2.**
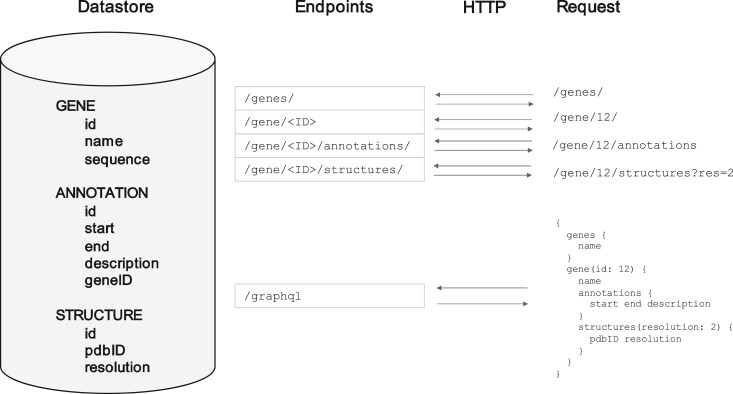
An example of a simple bioinformatics dataset served using REST and GraphQL. Here, the underlying dataset is comprised of genes (each with a name and a sequence), each of which can have zero or more associated annotations and structure files. With REST, there are different endpoints offering access to different prestructured datatypes, such as all genes, the annotations for a particular gene and the structures for a particular gene. To build up a complete picture of all the annotations and structures for the genes, the client is forced to submit four requests. The API could be restructured to reduce this, but it would then have this particular use case in mind and would be ill-suited for some other situation. The GraphQL implementation by contrast does not try to anticipate the clients’ needs. There is a single endpoint, from which the client can obtain precisely the information it needs in one request. Also, note that in REST the client is forced to download the sequence information for each gene, whether it needs it or not

Figure 2 demonstrates an example of a simple bioinformatics dataset, comprising genes, gene annotations and gene product structures, and shows how an API might be built for this using both REST and GraphQL. In the latter case, a single GraphQL query is sent which asks for a specific instance of the Gene type and, because the schema defines the relationship between the three object types, the user can request information about these related objects in the same query, without the backend developer needing to anticipate any particular use case.

GraphQL has three fundamental operations. The first of these is the ‘query’, mentioned previously, which obtains data from the server without modifying anything on the server. The second is the ‘mutation’, a request for data which (where allowed) also alters something on the server, similar to REST verbs, such as POST, PUT and DELETE. The third is the ‘subscription’, a long-lived connection between client and server in which the server can push data back to the client when it is ready, rather than the client having to request it. This can be used for large data or where the process of generating the data takes a long time and cannot be done in a single request/response cycle.

The advantages of GraphQL are its flexibility, and the power it grants to the client. With REST and SOAP, the kinds of responses that can be returned are fixed, finite, inflexible and chosen by developers in an attempt to anticipate the needs of the client. The client combines this small number of possible responses to obtain the required data. However, the client generally ends up also obtaining data that were not asked for (e.g. only one attribute of an object may have been needed). If the client is requesting data about different related object types, it has to make multiple requests to retrieve them. These problems are referred to as over-fetching and under-fetching, respectively, and result from the fundamental limitation of both REST and SOAP—they force API developers to try to anticipate the use cases of the API and design for those. Even if they anticipate correctly, these will still be a small subset of the possible requirements of the client. In contrast, with GraphQL, the API developer simply defines the relationships between the different object types and gives the client the ability to request precisely what it needs. The API developer does not need to anticipate the needs of the client—the client requests what it needs, and nothing more.

It should be noted, however, that the ‘graph’ in GraphQL refers to the graph of object *types*—the data themselves are returned as JSON, a tree-like structure which is not especially well suited to representing datasets which are themselves cyclic graphs, such as those from graph databases like neo4j. There have been recent attempts to define a standard language for representing these graphs using GraphQL (Hartig and Hidders, 2019).

GraphQL was developed by Facebook in 2012 (Facebook, 2015), in response to REST’s inability to represent the complex interconnected and recursive objects used in the social network properly and efficiently. It was publicly released as a specification in 2015 (Facebook, 2018) and has seen gradual and sustained adoption. GitHub chose to employ GraphQL for version 4 of its web API in 2016, alongside its older REST API, citing its ‘powerful advantages over REST’ (GitHub, 2018). Since then, Twitter has rebuilt its public API in GraphQL, as have a number of other prominent technology companies. Just as there was a shift from SOAP to REST, recent years have seen the beginnings of a move away from REST to GraphQL. In addition to the reference implementation in JavaScript, there are libraries for quickly generating GraphQL backends in Python [graphene (Akbary, 2020)], Perl, Java, C, C++, C#, Julia and others (https://graphql.org/code/).

### 1.2 GraphQL and bioinformatics

To date, however, GraphQL has seen limited use in bioinformatics contexts. This is surprising, not only because of the advantages already outlined here but also because, in many respects, GraphQL is *particularly* well suited to the needs and typical characteristics of bioinformatics resources. There are currently just a small number of examples. gnomAD (Wang *et al.*, 2020), a database of genome sequences aggregated from multiple sources, has recently implemented a GraphQL API and the RCSB has provided a GraphQL endpoint since early 2020 in addition to its REST API (https://www.rcsb.org/pages/webservices). Generally, however, REST APIs remain by far the most common.

Of particular interest for bioinformatics is the GraphQL subscription operation. Computationally intensive jobs (asynchronous tasks that cannot be completed within the time-frame of an HTTP request/response cycle) are a common feature of bioinformatics (Stockinger *et al.*, 2008). Web interfaces often use page redirection or AJAX (asynchronous JavaScript and XML) while APIs typically require the client to be sent a job ID of some sort as a response, with which the client must then poll the server continuously to obtain the status of the job and, when complete, obtain the results. If the amount of data being requested is large, this is wasteful of network resources and, depending on the interval between requests, means there will usually be a delay between the job completing and the client learning of this and obtaining the output. With GraphQL, the client can initiate the job using a subscription, and a connection between server and client is then maintained using a WebSocket rather than HTTP. No constant back and forth chatter is required as the server will immediately push the output to the client once ready. This could greatly simplify the way bioinformatics services are accessed in a web browser, where APIs for using websockets are already available, though outside the browser they do require additional libraries that can support this separate protocol.

The often-complex nature of bioinformatics datasets, particularly their complex internal relationships, is also well-served by modelling them as a graph. Any hierarchy and any level of nesting can be explored and requested in a single GraphQL request, rather than the multiple requests to different REST endpoints that would be required otherwise, as demonstrated in Figure 2. Rather like SOAP, the GraphQL schema makes GraphQL APIs explorable and self-documenting in a way that REST APIs are not. This is of considerable importance in a bioinformatics context, where entities tend to be numerous and interconnected, and the structure of the corresponding API that describes those data is not intuitive or predictable in the absence of documentation. An example is given below of our own ZincBindDB API, which has 10 distinct object types with fields pointing to each other, and many bioinformatics resources have much more complex hierarchies of entities than this. Representing these relationships as an arbitrarily queryable GraphQL schema rather than a rigid REST API makes the dissemination of biological data easier.

Thus, while GraphQL can offer an improved API experience for web resources in general, bioinformatics web resources would be particularly well served by increased adoption.

## 2 Methods

### 2.1 A GraphQL interface to ZincBind

ZincBind, the database of zinc-binding sites (Ireland and Martin, 2019), comprises ZincBindDB (the continuously updated database itself, containing zinc-binding sites collected from the Protein Data Bank and organized into groups and families) and ZincBindPredict (Ireland and Martin, 2021) (predictive models of zinc binding for structure and sequence). Recently, we replaced ZincBind’s initial REST API with a GraphQL API, using the Python library Graphene (Akbary, 2020). This was a straightforward task, as the existing Django database objects translate well to GraphQL object types—in most cases, the GraphQL types are generated automatically from the database tables using Graphene’s plugins for mapping Django tables and fields to GraphQL types and fields, respectively. The same plugins also automatically generate resolvers for populating queries with data using database lookups. Consequently, these automatically generated types do not need to have resolvers written for them, unless custom logic is required. This allows a GraphQL schema to be generated from the database schema, without having to define the fields and resolver functions manually for each type.

**Fig. 3.**
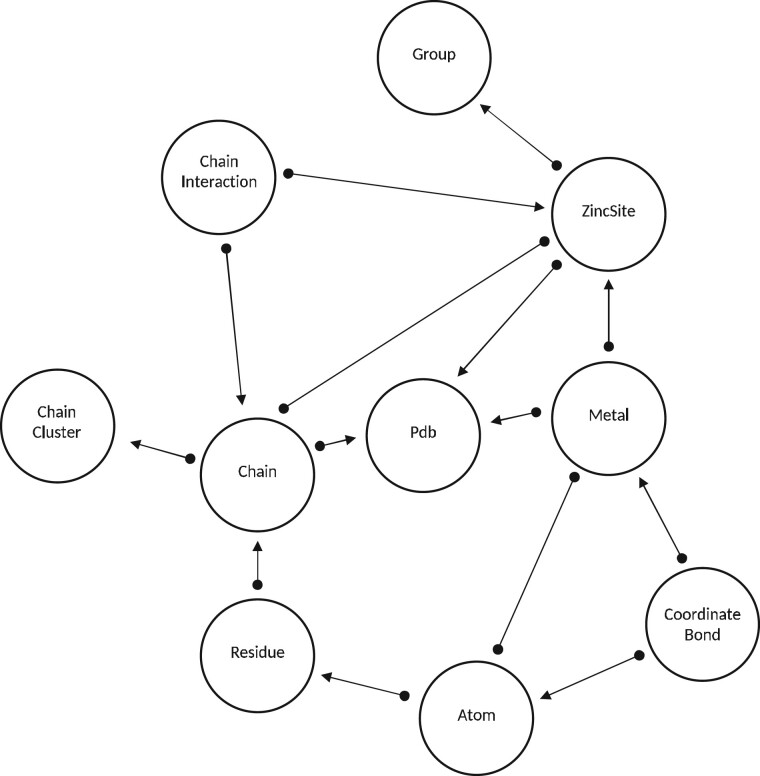
A graph of the object types in ZincBind, as represented by GraphQL. Each circle is an object type node, and the arrows are the edges between them. Here, a circle at the end of an edge represents multiple object types, and an arrow represents a single object type—for example, one chain has many residues, one residue has many atoms, etc. Such a complex network of inter-related object types is typical of bioinformatics resources and is poorly served by a REST API. Not shown here are the primitive types, the Query object type which has edges with all object types or the Connection object types used to facilitate pagination. The complete schema is shown in Supplementary File S1

The ZincBindDB API is available at https://api.zincbind.net, and is a read-only data access portal for the database, supporting only queries. The user submits GraphQL queries, for which the different object types of PDB, ZincSite, Residue, Atom, Chain, etc. are all accessible, with their relationships defined, such that any combination of relationships can be explored in a single query. The object types are related to each other in a reasonably complex network of one-to-many relations, as shown in Figure 3. Such a network of object types is difficult to represent using REST, as we had to select what we imagined to be the most common object pairs and create specific endpoints for representing those. With GraphQL, no such assumptions need to be made—the user can create a single request to explore any set of relationships they wish. (The full schema is available in text form in Supplementary File S1).

We found that the sheer flexibility of GraphQL required certain minimal precautions to be taken with regard to nesting. GraphQL allows recursive nesting of object types (e.g. requesting all the zinc sites within a PDB entry, every chain associated with each of those zinc sites, every zinc site associated with each of those chains and so on) which, by default, can increase the number of underlying SQL queries made on the server exponentially with every layer. This can be ameliorated by imposing a limit on nesting, or (as is the case in our implementation) using intelligent caching of database calls to ensure needless duplicate SQL queries are not made.

ZincBindPredict, our associated zinc-binding site prediction tool, accepts jobs in the form of a GraphQL mutation. The client can submit a protein sequence using the ‘searchSequence ’ mutation or a protein structure using the ‘searchStructure ’ mutation, with the latter using the GraphQL Multipart Request Specification for uploading the coordinate file (an extension to the GraphQL specification that allows files to be sent over HTTP requests alongside the query/mutation). Coordinates can be contained in any filetype supported by the atomium protein structure Python library (Ireland and Martin, 2020). As with long-running REST calls, the API will return a job ID, which the client can then use to submit queries to obtain the status of the job and, when complete, the list of rejected or predicted residue combinations for a given binding site family. Given the asynchronous model employed here, using a subscription rather than a mutation would also have been a valid design, but we opted for a mutation owing to the relatively small amount of data being delivered to the client. The use of a mutation also avoids the need for the client to use a websocket library making client implementation more straightforward.

The flexibility of GraphQL responses is particularly useful here. In predicting zinc-binding sites, there tends to be a very large number of rejected residue combinations, and a small number of combinations that are predicted to be binding sites. Having options to obtain the list of rejected sites, not to include these in the response, or just to request their count, all without having to define separate endpoints as one would with REST, eases the burden on the network. For example, the ZincBindWeb frontend to both ZincBindDB and ZincBindPredict consumes the GraphQL APIs and does not request the rejected sites.

**Fig. 4.**
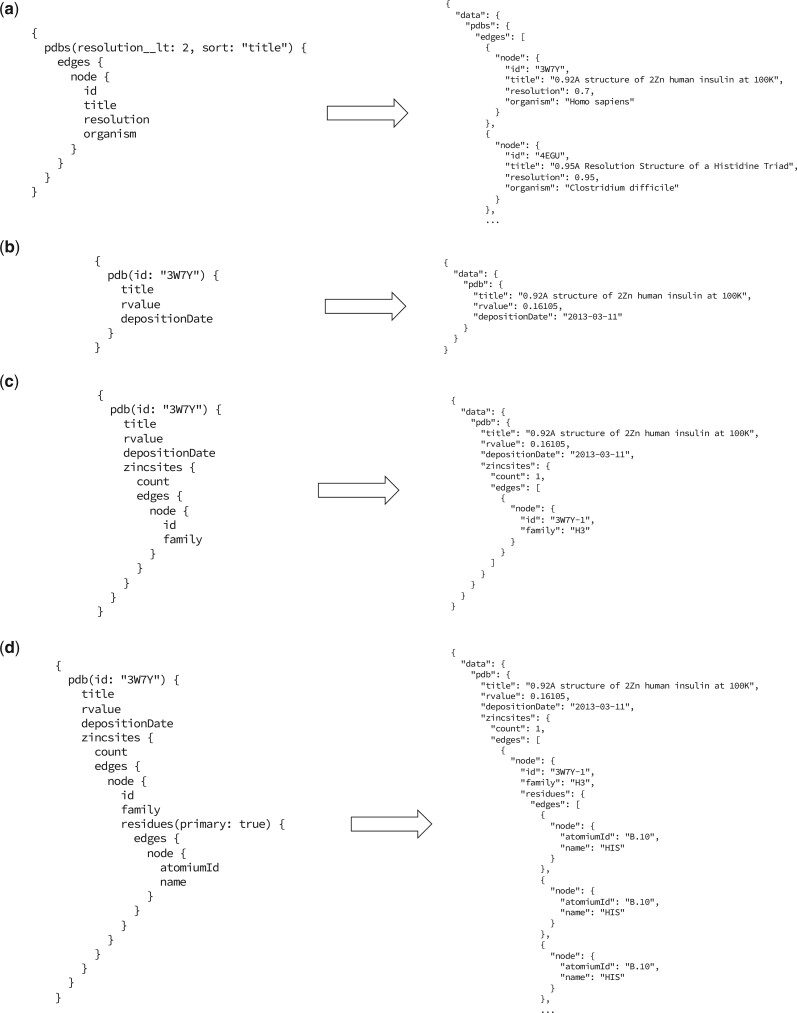
(**a**) A query requesting a list of PDB objects with a resolution better than 2, sorted by title. These qualifiers are given as arguments to the pdbs field of the root query object. Note that while it is possible to return lists as direct children of the parent object (Query in this case); here, the common convention of having pdbs be a connection object containing meta-information about the list (such as count), which in turn has a list of edge objects with pagination cursors, which each map to a single PDB object. This model, though more complex for a beginner, is a common way of representing lists in web applications. Note also that the structure of the JSON response matches the structure of the query. (**b**) A query requesting a single PDB object, by an ID given as an argument. Here, a different subset of fields is requested, which in this case are all primitive types. While these examples all use this PDB object, other objects in the schema can also be requested in this way—see Supplementary File S1 for the full schema. The rvalue here is a ‘nullable’ field, in that it can return null in some cases. (**c**) A query for the same PDB object, but here requesting all the zinc-binding sites within that PDB—there is one in this case. In this case, the field’s id and family are requested for each, though they also have other fields. (**d**) The same query, but this time requesting all primary residues for every binding site (rather than all residues, which would include second shell residues). GraphQL queries can be nested indefinitely, though care should be taken when implementing resolvers such that recursive queries do not create large numbers of database queries

Practical examples of ZincBindDB API queries are given in Figure 4, which illustrates the general principles of GraphQL queries, as well as the specifics of how this API is structured. Figure 4a and b illustrates how one would obtain multiple instances of an object (in this case PDB entry objects with resolution <2.0 Å) and a single object (a PDB object with a specific identifier). Both pdbs and pdb are fields of the top-level Query object, with the latter representing a single PDB object, specified with an id argument, and the former representing a list of PDB objects which can be filtered by property. Figure 4c and d demonstrates how queries can be nested, requesting information about three interconnected objects (PDB, ZincSite and Residue). In the case of Figure 4d, this is information that would be likely to require multiple requests with REST.

**Fig. 5.**
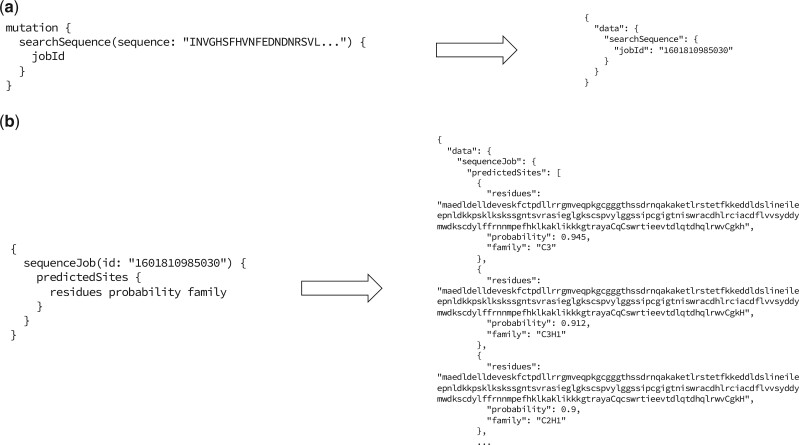
(**a**) A mutation from the ZincBindPredict API. Mutations begin with the mutation identifier to indicate that the top-level Mutation’ object is what the ‘searchSequence’ mutation belongs to—queries can begin with ‘query’ too, but this is optional. Here, the sequence is being given as an argument, and the ID of the job is returned. (**b**) A query for the results of a ZincBindPredict job. The ID of the job is supplied, and this particular query requests the status of the job, as well as the predicted sites. The rejected sites can also be requested, but since they are quite numerous, typically the flexibility of GraphQL in allowing one to choose to omit them is useful in conserving network resources

The job system for the ZincBindPredict API is illustrated in Figure 5. Figure 5a shows how a sequence prediction job is submitted to ZincBindPredict using a GraphQL mutation—these are similar to queries except that their resolver functions will have side effects of some kind. In this case, that side effect is to start a job running on the server. Structure prediction jobs work in much the same way, except that a file is uploaded using the GraphQL Multipart Request specification instead. Figure 5b shows a more straightforward query using the previously obtained job ID to fetch information about the job—crucially, only the information the client needs. As described above, this could also be implemented using a GraphQL subscription.

## 3 Discussion

Bioinformatics resources have followed, and often led, trends in the computing world generally in the consolidation of web services around SOAP and REST, with the latter coming to predominate. The recent development of an alternative mode of supplying web services, the graph-oriented GraphQL specification, offers unique advantages to bioinformatics web services, such as a subscription model for updating the result of complex operations, and response-size management through specifying particular fields and relations to return. Here, we have also shown two concrete examples of such an implementation for both traditional data dissemination, and for running time-consuming jobs for predicting zinc binding. Changing from REST to GraphQL in ZincBind has made the ZincBind website (which consumes its own API) much easier to develop and will make future enhancements considerably easier.

## Funding

This work was supported by a Wellcome Trust PhD Studentship to S.M.I. [reference: 203756/Z/16/A].

*Conflict of Interest*: none declared.

## Data availability

All data and code are available at https://api.zincbind.net and https://github.com/samirelanduk/ZincBindWeb

## Supplementary Material

vbab023_Supplementary_Data
